# Comparisons of artificial intelligence algorithms in automatic segmentation for fungal keratitis diagnosis by anterior segment images

**DOI:** 10.3389/fnins.2023.1195188

**Published:** 2023-06-08

**Authors:** Dong-Jin Li, Bing-Lin Huang, Yuan Peng

**Affiliations:** ^1^Health Management Center, The First People's Hospital of Jiujiang City, Jiujiang, Jiangxi, China; ^2^College of Clinical Medicine, Jiangxi University of Traditional Chinese Medicine, Nanchang, Jiangxi, China; ^3^Department of Ophthalmology, The Affiliated Hospital of Jiangxi University of Traditional Chinese Medicine, Nanchang, Jiangxi, China

**Keywords:** anterior segment images, artificial intelligence, automatic segmentation, fungal keratitis, diagnosis

## Abstract

**Purpose:**

This study combines automatic segmentation and manual fine-tuning with an early fusion method to provide efficient clinical auxiliary diagnostic efficiency for fungal keratitis.

**Methods:**

First, 423 high-quality anterior segment images of keratitis were collected in the Department of Ophthalmology of the Jiangxi Provincial People's Hospital (China). The images were divided into fungal keratitis and non-fungal keratitis by a senior ophthalmologist, and all images were divided randomly into training and testing sets at a ratio of 8:2. Then, two deep learning models were constructed for diagnosing fungal keratitis. Model 1 included a deep learning model composed of the DenseNet 121, mobienet_v2, and squeezentet1_0 models, the least absolute shrinkage and selection operator (LASSO) model, and the multi-layer perception (MLP) classifier. Model 2 included an automatic segmentation program and the deep learning model already described. Finally, the performance of Model 1 and Model 2 was compared.

**Results:**

In the testing set, the accuracy, sensitivity, specificity, F1-score, and the area under the receiver operating characteristic (ROC) curve (AUC) of Model 1 reached 77.65, 86.05, 76.19, 81.42%, and 0.839, respectively. For Model 2, accuracy improved by 6.87%, sensitivity by 4.43%, specificity by 9.52%, F1-score by 7.38%, and AUC by 0.086, respectively.

**Conclusion:**

The models in our study could provide efficient clinical auxiliary diagnostic efficiency for fungal keratitis.

## Introduction

Fungal keratitis, also known as keratomycosis, is a common blinding eye disease (Thomas et al., 2005). The main manifestations are corneal infiltration, rough corneal edge, and “satellite” lesions (Mahmoudi et al., 2017). Patients often suffer from eye injury, require eye surgery, must wear contact lenses, and suffer from other diseases caused by organic substances (especially plants; Ali Shah et al., 2017). According to statistics, every year ~1–14 million people are infected with fungal keratitis worldwide, of which 75% of patients might be blind in one eye and 60% of patients might be blind even after treatment (Brown et al., 2021), which results in a huge burden to families and society. Therefore, early diagnosis and treatment of fungal keratitis is necessary. However, at present, fungal keratitis diagnosis depends mainly on traditional microbial culture (Sadik et al., 2022), which takes considerable time and cannot provide a basis for early treatment. At present, the diagnosis of fungal corneal ulcer is mainly based on confocal microscopy of corneal culture. Fungal corneal ulcer can cause corneal perforation and fungal endophthalmitis. Thus, accurate and rapid early diagnosis of fungal keratitis is important.

Recently, artificial intelligence (AI), especially machine learning (ML), has been applied in the field of ophthalmology (Lee et al., 2020) and has a significant role in corneal disease diagnosis (Siddiqui et al., 2020). A corneal ulcer can be diagnosed by anterior segment photography. At the same time, artificial intelligence technology has shown better diagnostic efficiency in medical image processing. Moreover, ML based on the deep neural network (DNN) is called deep learning and is considered the most advanced ML (LeCun et al., 2015; Litjens et al., 2020). Huang et al. used the deep learning model built by different convolutional neural networks (CNNs) to evaluate 580 patients to help distinguish bacterial keratitis (BK) and fungal keratitis quickly in clinical practice and found that DenseNet 161 in CNN has the best performance. This deep learning model can improve the recognition rate significantly between the two kinds of keratitis and provide better accuracy for clinical diagnosis (Hung et al., 2021). Additionally, Li et al. compared the classification ability of AlexNet, DenseNet 121, and InceptionV3 algorithms for 48,530 slit lamp images of different keratitis and found that DenseNet 121 had the best classification performance (Li et al., 2021). AI is used widely in the field of keratitis diagnosis, and algorithms, such as DenseNet 161 and DenseNet 121, have high performance in deep learning models. However, most of the existing AI-assisted diagnosis of fungal keratitis methods compare the performance of different single algorithm models. The application of the deep learning model built by integrating these different algorithms in the diagnosis of keratitis is relatively rare. In contrast to the abovementioned research, Ghosh et al. used three deep learning models constructed by VGG19, DenseNet 121, and RestNet50 to separate fungal keratitis and BK and then compared the results of each model and ensemble learning. Finally, ensemble learning had the largest area under the precision-recall curve (AUPRC) compared with any single architecture model, and they believed that ensemble learning can improve the performance of assisted diagnosis of diseases significantly (Ghosh et al., 2022). Therefore, the ensemble learning model composed of multiple algorithms is more accurate. Ensemble learning is a kind of fusion technology that is a fusion at the model level and belongs to late fusion. Early fusion is also named feature-level fusion, which emphasizes the data combination before the classification. The final feature vector consists of the features extracted from heterogeneous signals, and early fusion should put the final feature vector into the classifier alone (Zhang et al., 2017).

Currently, prior AI studies have mainly focused on the diagnosis of viral keratitis and bacterial keratitis. Most previous studies used traditional machine learning or deep learning based on original slit lamp images. No studies have investigated the early fusion method for fungal keratitis. Moreover, previous studies were based mostly on whole anterior segment images. However, the area outside the keratitis lesion might affect the performance of models. Therefore, it is necessary to segment the lesion area from the images. Manual segmentation is tedious, time-consuming, and user-dependent (Wang et al., 2016), and automatic segmentation can be faster but might not have the same accuracy as manual segmentation (Wang et al., 2016; Huang et al., 2019). Thus, we hypothesized that the early fusion method for fungal keratitis with automatic and manual segmentation may show better diagnostic and sorting efficiency.

Therefore, this study combines automatic segmentation and manual fine-tuning with an early fusion method to provide efficient clinical auxiliary diagnostic efficiency for fungal keratitis. In detail, we developed two AI platforms with a deep transfer-learning algorithm and multi-feature fusion for fungal keratitis and non-fungal keratitis; one is based on an automatic segmentation method, whereas the other is based on a manual segmentation method.

## Materials and methods

### Study design

**Figure 1:**
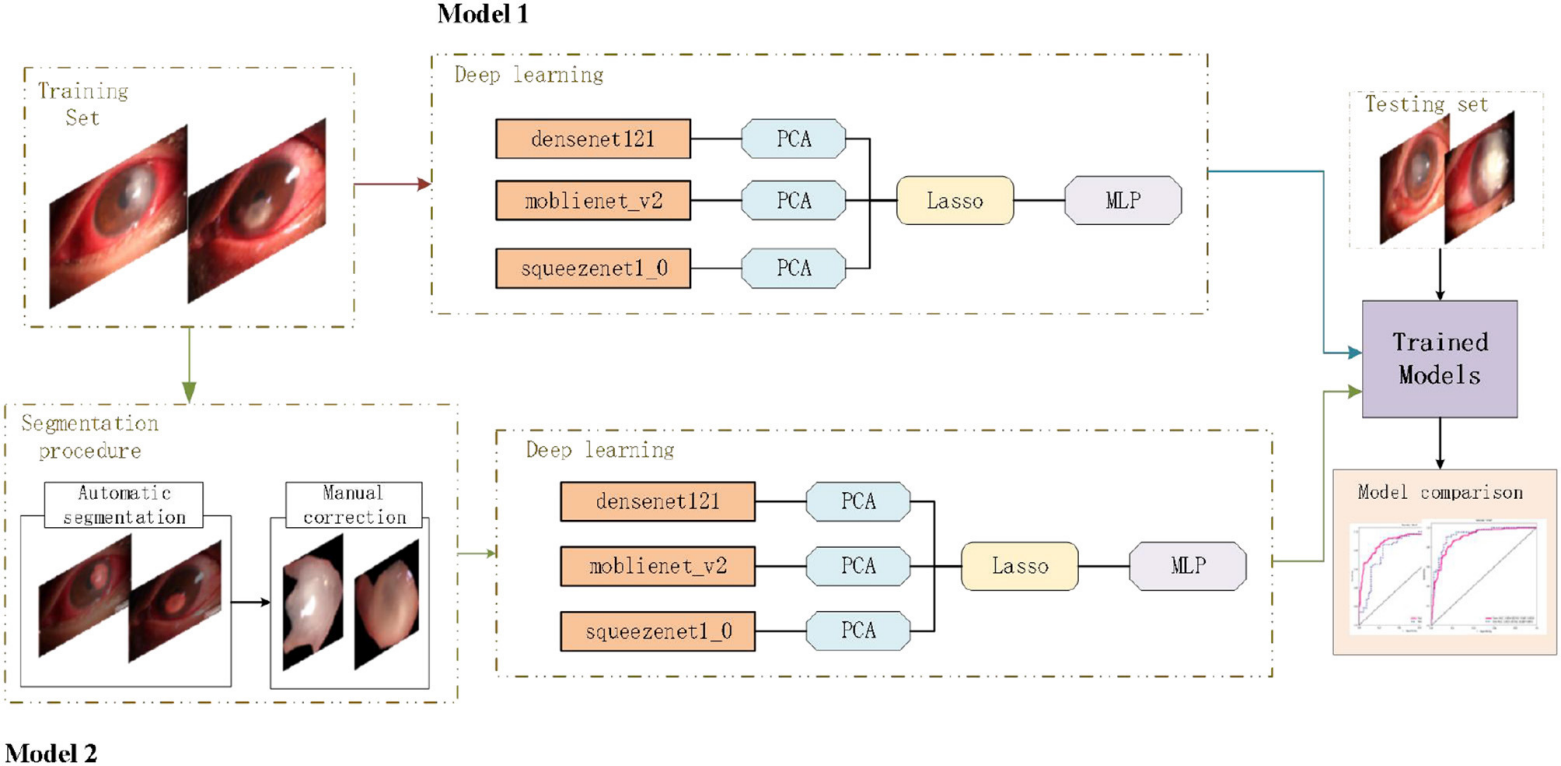
Deep learning pipeline. The two deep learning models were developed separately, and the performances of different models were compared based on a new testing set.

To realize the automated diagnosis of fungal keratitis, two deep learning models were constructed. Model 1 only included a deep learning model which was composed of the DenseNet 121 (The idea of Dense Connection is used, that is, every layer is connected with all the previous layers, so that the model has better information transmission and reuse ability in feature extraction. DenseNet 121 refers to the fact that the model has 121 layers), mobienet_v2 (this is a lightweight Convolution neural network model, mainly is the depth of Separable Convolution (Depthwise Separable Convolution) and Linear Bottleneck (Linear Bottleneck) technology, such as small parameters, run fast) and squeezentet1_0 models (Squeezentet1_0 is another lightweight convolutional neural network model, which is composed of a Squeeze layer and an Expand layer. It also runs fast with fewer parameters) which are common convolutional neural network models are used for image classification and object detection. The least absolute shrinkage and selection operator (LASSO) model and multi-layer perception (MLP) classifier. Model 2 included the automatic segmentation program and the deep learning model as described above. The deep learning pipeline of our study is shown in Figure 1.

### Establishment of a dataset and image preprocessing of anterior segment images

We collected 423 high-quality anterior segment images of keratitis in the Department of Ophthalmology of the Declaration of Helsinki and were approved by the Medical Ethics Committee of the affiliated Hospital of Jiangxi University of Traditional Chinese Medicine from February 2020 to September 2023. The inclusion criteria of fungal keratitis are as follows: the corneal scrape was examined with 10% potassium hydroxide wet tablet bacteria or cornea, necrotic tissue, and pus in the potato culture medium to see bacteria falling growth and to make clinical manifestations such as ulcer surface with moss-like bad dead tissue, satellite foci, feathery edges, and furrow pits that can be seen around ulcer depression, and focal stromal infiltration dense, that may be accompanied by stromal abscess. The cornea is often pasted with a white mushy posterior corneal deposit (KP), anterior room pus color white matter thick, longer use of antibiotics, or cortical stimulation of patients with ineffective vegetarian treatment or ulcer aggravation.

To protect patient privacy, identifiable information was removed. Then, the images were divided into the fungal keratitis group and non-fungal keratitis group by a senior ophthalmologist, and all the images were randomly divided into training and testing sets at the ratio 8:2. The dataset contained a training set and a testing set, where the training set contained 168 fungal keratitis images and 170 non-fungal keratitis images, and the testing set contained 42 fungal keratitis images and 43 non-fungal keratitis images. This study was approved by the Ethics Committee of Jiangxi Province Peoples Hospital and adhered to the Declaration of Helsinki and the ARVO statement on human subjects.

### Establishment of the automatic segmentation model

First, based on the anterior segment images, a senior ophthalmologist used the LabelMe software (https://github.com/wkentaro/labelme) to annotate the keratitis lesions area as the region of interest (ROI), respectively. The ROI of each image was annotated as “label 0” or “label 1.” “Label 0” was defined as the fungal keratitis lesions area. “Label 1” was defined as the non-fungal keratitis lesions area. The FCNResnet50 which was a fully convolutional network based on ResNet50 was used to extract the ROI masks. First, the original images in the training set were used to train the FCNResnet50 model, and the obtained optimal parameters were then applied to the whole anterior segment images to get the automatic segmentation mask. Then, the manual segmentation mask annotated by the senior ophthalmologist was used as the gold standard. After the segmentation errors were adjusted, the final mask was obtained. Based on the final masks, the keratitis lesions area was segmented.

### Establishment of the deep learning diagnostic model

The fungal keratitis detection was defined as a binary classification problem, with a label of 0 or 1 indicating that the image was fungal keratitis or non-fungal keratitis. This classification task was performed by the deep learning diagnostic models composed of DenseNet 121, mobienet_v2, squeezentet1_0, the least absolute shrinkage and selection operator (LASSO) model, and the multi-layer perception (MLP) classifier. In the training set, first, we used three models to extract features of the penultimate layers of the network and principal components analysis (PCA) in feature dimensionality reduction. Then, the features after dimensionality reduction were fusioned by channel concat which meant that the layer stacked features from each branch together. The LASSO logistic regression algorithm was used to select the optimal features. Finally, the optimal feature set was input into the MLP classifier to establish the final diagnostic model. In the testing set, the 5-fold cross-validation was performed for parameter optimization. The selected features and the best parameters were applied for model evaluation.

### Comparison and validation of diagnostic models

In Model 1, the original images in the training set were used to train the deep learning diagnostic model, and the original images in the testing set were used to validate this model. In Model 2, first, the original images in the training set were used to train the automatic segmentation model, then after manual fine-tuning, the keratitis lesions area segmented from the original images which were in the training set was used to train the deep learning diagnostic model. Finally, the original images in the testing set were used to validate Model 2.

To compare the performance of the two models, the receiver operating characteristic (ROC) curve was performed in this study to analyze the diagnostic ability of each model. The decision curve analysis (DCA) was used to evaluate the net benefit of the models for clinical decisions. The highest curve at any given threshold probability is the optimal decision-making strategy to maximize the net benefit (Gao et al., 2022). The gradient-weighted class activation mapping (Grad-CAM) was used for the visual verification of the diagnostic results of this method. The heatmap images created by the Grad-CAM indicated where the deep learning model was focused.

### Statistical analysis

For the automatic segmentation model, we used the pixel-level classification accuracy, the average intersection-over-union (IOU), and dice coefficient to evaluate the performance. The pixel-level classification accuracy was the percentage of correctly classified pixels out of the total pixels in each image, and IoU evaluated precision by calculating the overlap between the prediction and target variables (Mahmoudi et al., 2017; Larsen et al., 2021). The dice coefficient is a set similarity measure function, the higher the dice coefficient, the better the segmentation effect (Li et al., 2020). For the deep learning diagnostic model, we measured the accuracy, sensitivity, specificity, and F1-score from the training set and testing set. We also plotted the DCA curves and the ROC curves from the two models. The area under the curve (AUC) with a 95% confidence interval (95% CI) of each model which was in the training set and testing set was calculated. All the methods were implemented in Python language using Python 3.9.7 version.

## Results

### Performance of automatic segmentation model

**Figure 2:**
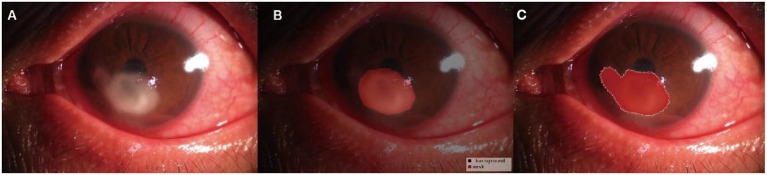
Diagram of the segmentation image effect of the keratitis lesions area: the original images **(A)**, the automatic segmentation results **(B)**, and the manual segmentation result **(C)**. The red area indicated the segmented lesion area of keratitis.

The pixel-level classification accuracy was 96.2%. The average IoU score was 81.3%. The mean dice score was 89%. The diagram of the segmentation image effect of the keratitis lesions area is shown in Figure 2.

### Comparing the different deep learning diagnostic models in diagnosing fungal keratitis

In the testing set, the accuracy, sensitivity, specificity, and F1-score of Model 1 reached 77.65, 86.05, 76.19, and 81.42%, respectively. For Model 2, which is based on the segmentation images, the accuracy improved by 6.87%, sensitivity by 4.43%, specificity by 9.52%, and F1-score by 7.38%, respectively, as shown in Table 1.

**Table 1:** Performance comparison of Model 1 and Model 2.

**Model name**	**Train/test**	**Accuracy**	**AUC**	**95% CI**	**Sensitivity**	**Specificity**	**F1-score**
Model 1	Train	82.84%	0.905	(0.874–0.937)	82.94%	83.33%	82.94%
	Test	77.65%	0.839	(0.751–0.927)	86.05%	76.19%	81.42%
Model 2	Train	81.71%	0.894	(0.861–0.928)	85.38%	79.76%	83.21%
	Test	84.52%	0.925	(0.869–0.981)	90.48%	85.71%	88.80%

**Figure 3:**
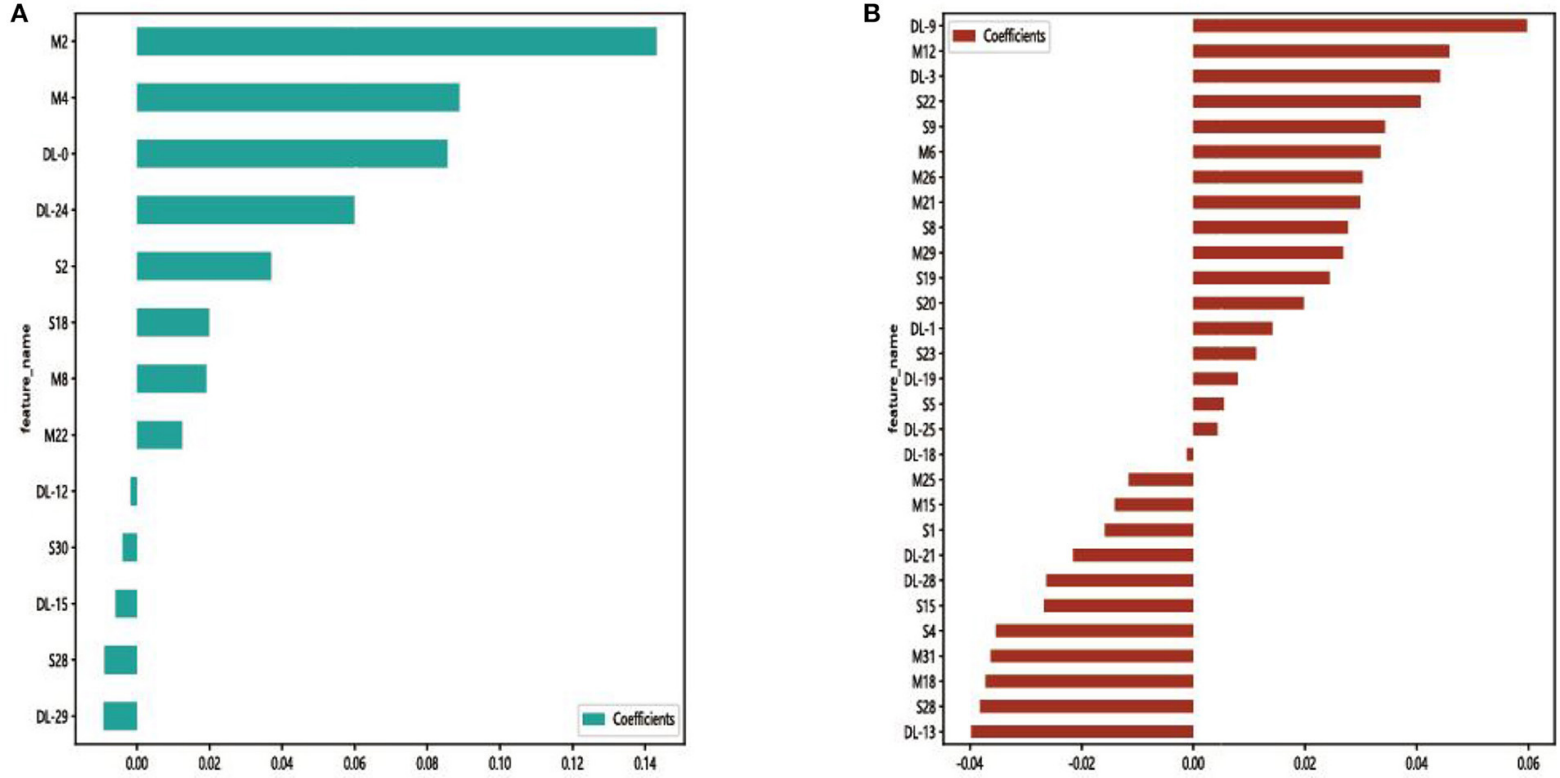
Selected features and their coefficient values in Model 1 **(A)** and Model 2 **(B)**. DL, M, and S indicate that the feature was from the DenseNet 121, mobienet_v2, and squeezentet1_0 models, respectively.

**Figure 4:**
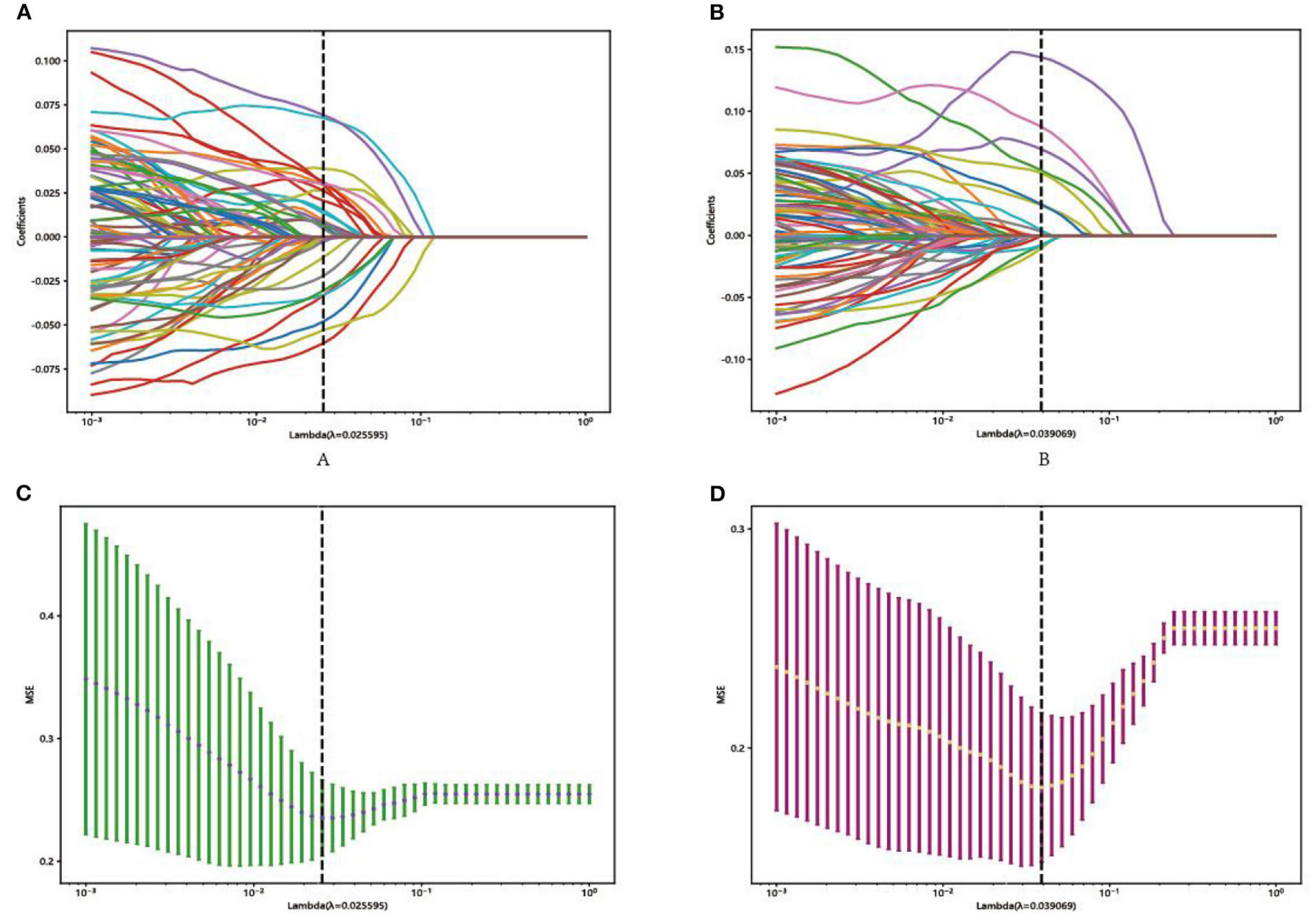
Representative LASSO coefficient distribution map, Model 1 **(A)** and Model 2 **(B)**. Selection of features based on the LASSO regression model, Model 1 **(C)**, and Model 2 **(D)**.

In each model, a total of 50,176 features, 62,720 features, and 43,265 features were extracted from the DenseNet 121, mobienet_v2, and squeezentet1_0 models separately. After dimension reduction and channel concat, a total of 93 features were retained. After screening with the Lasso model, 13 features and 29 features were left for further classification in Model 1 and Model 2, respectively. The selected features and their coefficient values are shown in Figure 3. The LASSO screening process is shown in Figure 4.

**Figure 5:**
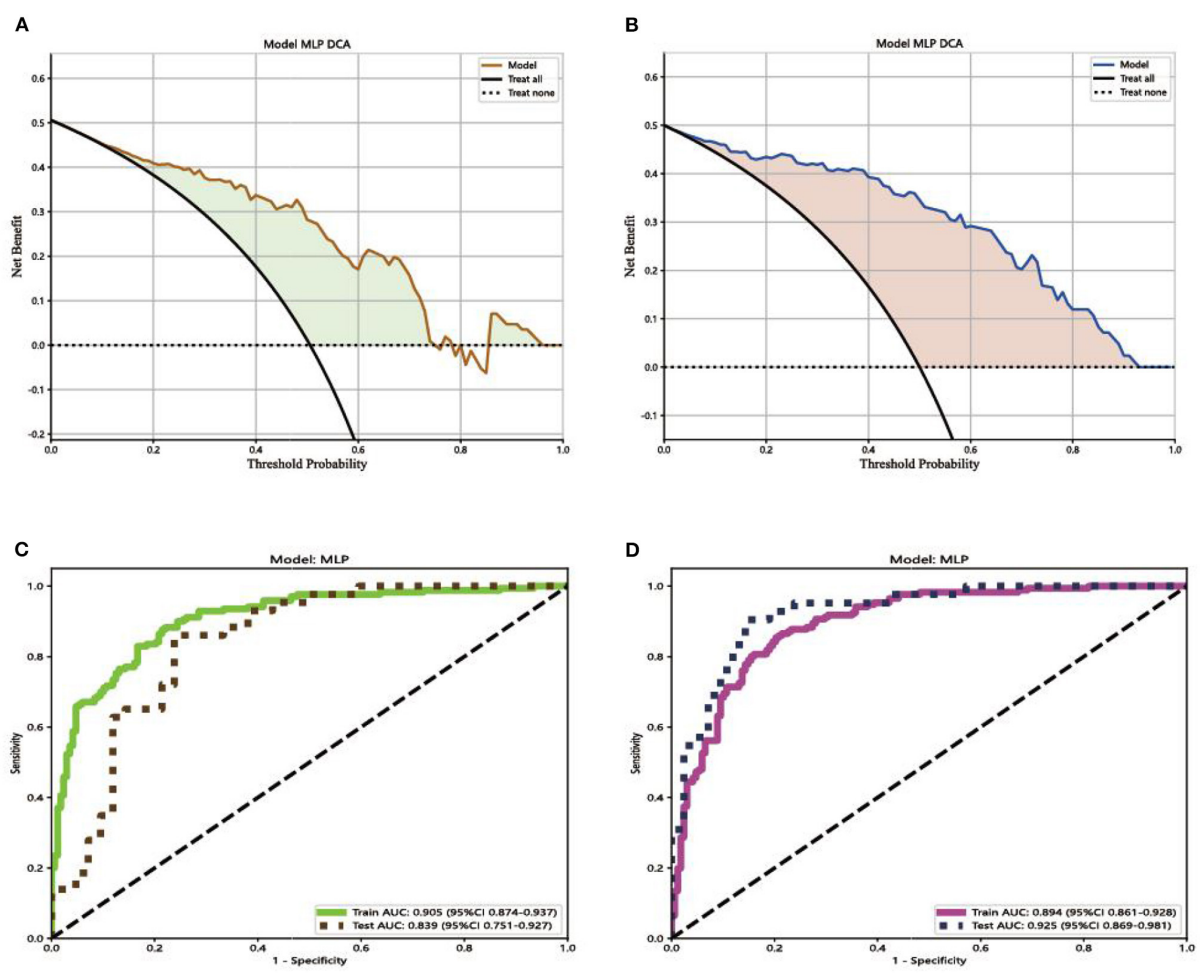
Decision curves and receiver operating characteristic (ROC) curves for the different models. **(A)** The net benefit of Model 1 in making a correct diagnosis of fungal keratitis; **(B)** the net benefit of Model 2 in making a correct diagnosis of fungal keratitis. The x-axis is the threshold probability, and the y-axis measures the net benefit. “Treat none” indicates that all samples were negative without intervention and the net benefit was 0. “Treat all” indicates that all samples were positive with intervention. **(C)** ROC curve for the different Model 1; **(D)** ROC curve for Model 2. AUC indicates the area under the curve of ROC.

Comparing the results of ROC curves, in the testing set, Model 1 achieved an AUC of 0.839 (95% CI 0.751–0.927). Model 2 achieved the highest AUC of 0.925 (95% CI 0.869–0.981). Compared with the result of the DCA curve analysis, Model 2 would substantially benefit in diagnosing fungal keratitis when the threshold probability was between 0 and 90% in the test set, which received a higher net benefit than Model 1, as shown in Figure 5.

### Visualization of the deep learning process

**Figure 6:**
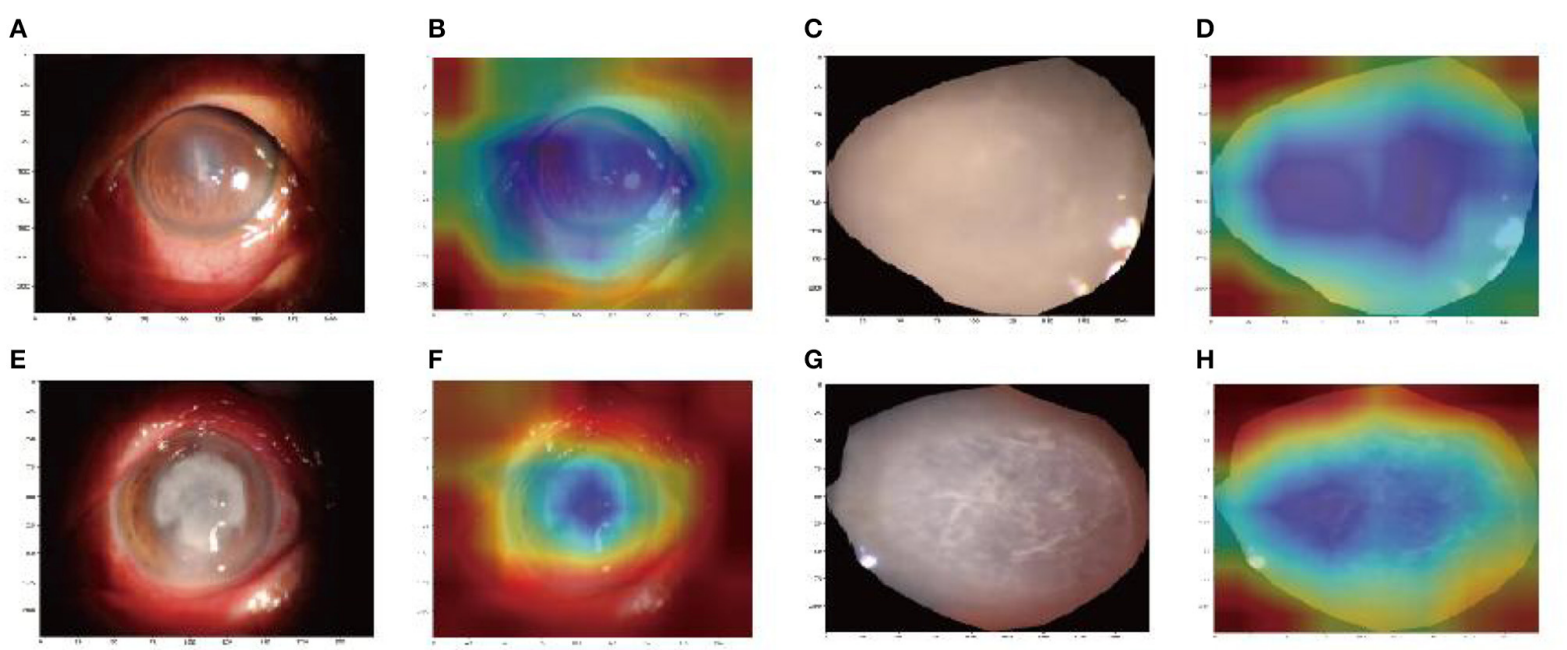
Image region heat maps based on Grad-CAM: the original images of Model 1 **(A, C)** and Model 2 **(E, G)**. The heat maps correspond to the original image of Model 1 **(B, D)** and Model 2 **(F, H)**. The blue areas were that the models likely focused on.

We used Grad-CAM to locate the important region for the classification. The results of the heat map displayed the areas which Model 1 likely focused on and were located in the keratitis lesions area but covered the surrounding normal corneal tissues. The areas that Model 2 likely focused on were located in the center of the keratitis lesions area, as shown in Figure 6.

## Discussion

Delayed diagnosis remains the main reason for the poor prognosis of deteriorating lesions (Wei et al., 2023). Therefore, this study developed an automatic diagnosis model to provide efficient clinical auxiliary diagnostic efficiency for fungal keratitis. As far as we know, this is the first AI-assisted diagnostic model that combines automatic segmentation and manual fine-tuning with an early fusion method for fungal keratitis (FK) diagnosis.

Comparing the performance of Model 1 with Model 2 in our study, the accuracy, sensitivity, specificity, F1-score, and AUC of Model 2 were all significantly higher than that of Model 1 in the test set. In previous research, Hung et al. (2021) used U square Net (U^2^ Net) to crop the image of the cornea and various CNN for identifying BK and FK. The DenseNet 161 model had an accuracy of 65.8%, which was the highest among all the models. The performance of their models is far below ours, indicating the limitation of single features in classification (Geng et al., 2017). Zhang et al. (2022) used a CNN to classify infectious keratitis. The highest accuracy and AUC of individual models was 77.11%. After the fusion of ResNext101_32x16d and DenseNet 169 models, although the accuracy was improved by 0.6%, this result is still lower than that of our Model 2. This is probably owing to the precise segmentation of the keratitis lesion area in our model, which ruled out the interference of the background. The performance of Model 1 is lower than Model 2 in our study, which also confirmed this notion. From this, the performance of models could be further improved by combining the segmentation model with the fusion method.

The decision curve analysis was implemented to evaluate the clinical usefulness of the model for diagnosing FK. The decision curve of a model is compared with extreme cases that include all patients or none. A model can be recommended for clinical use if its net benefit is greater than treating all and no patients (Du et al., 2021). Comparing the result of Model 1 with Model 2, the two models were both better than extreme cases (none and all) in the test set. Model 2 has greater potential for clinical application. Comparing the results of ROC curves, in the testing set, Model 1 achieved an AUC of 0.839 (95% CI 0.751–0.927). Model 2 achieved the highest AUC of 0.925 (95% CI 0.869–0.981). Compared with the result of the DCA curve analysis, Model 2 would substantially benefit in diagnosing fungal keratitis when the threshold probability was between 0 and 90% in the test set. Through the multi-feature transfer learning method combined with an automatic or manual segmentation algorithm, the resulting automatic segmentation platform can diagnose FK more quickly, whereas the resulting manual segmentation platform can diagnose FK more accurately.

Another strength of this study is the Grad-CAM introduction. Deep learning models are usually regarded as black boxes because the information regarding which features are important cannot be interpreted easily from the model (Wang et al., 2019). In our study, the heatmap images of Grad-CAM highlighted the important areas in corneal ulcer images used for AI diagnosis, which interprets the deep learning process effectively.

Our study also has certain limitations. First, the sample size included in this study was small. Second, the diagnosis of fungal keratitis is not entirely accurate, and some subjects lack laboratory tests. Third, the study only diagnosed FK and did not distinguish between different types of keratitis. Finally, accuracy needs to be improved. Therefore, in future studies, we will attempt to introduce transform learning to identify different keratitis types.

## Conclusion

In this study, we combined automatic segmentation and manual fine-tuning with the early fusion method for FK diagnosis which provides efficient clinical auxiliary diagnostic efficiency for fungal keratitis. Through the multi-feature transfer learning method combined with an automatic or manual segmentation algorithm, the resulting automatic segmentation platform can diagnose FK more quickly, whereas the resulting manual segmentation platform can diagnose FK more accurately.

## Data availability statement

The raw data supporting the conclusions of this article will be made available by the authors, without undue reservation.

## Ethics statement

The studies involving human participants were reviewed and approved by the Medical Ethics Committee of the Affiliated Hospital of Jiangxi University of Traditional Chinese Medicine. The patients/participants provided their written informed consent to participate in this study. Written informed consent was obtained from the individual(s) for the publication of any potentially identifiable images or data included in this article.

## Author contributions

D-JL, B-LH, and YP contributed to data collection, conducted statistical analyses, and wrote the manuscript. All authors read and approved the final manuscript, contributed to the article, and approved the submitted version.
